# A real-world study of the efficacy and tolerability of fremanezumab in migraine patients with a median follow-up of 14 months

**DOI:** 10.1186/s42466-025-00395-y

**Published:** 2025-06-03

**Authors:** Shiho Suzuki, Keisuke Suzuki, Yasuo Haruyama, Hiroaki Fujita, Tomohiko Shiina, Saro Kobayashi, Mukuto Shioda, Ryotaro Hida, Koichi Hirata

**Affiliations:** 1 Department of Neurology, Dokkyo Medical University, 880 Kitakobayashi, Mibu, Shimotsuga, Tochigi 321-0293 Japan; 2 Integrated Research Faculty for Advanced Medical Sciences, Dokkyo Medical University, Mibu, Japan

**Keywords:** Migraine, Migraine days per month, Fremanezumab, Calcitonin gene-related peptide monoclonal antibody

## Abstract

**Background:**

To evaluate the long-term efficacy and safety of fremanezumab over a 2-year period in a real-world setting.

**Methods:**

This retrospective, observational, single-center cohort study included 165 patients with episodic migraine (EM) or chronic migraine (CM) who received fremanezumab treatment. The primary endpoint was the change in monthly migraine days (MMDs) from baseline to months 1–24. The secondary endpoints included changes in Migraine Disability Assessment (MIDAS) scores, adverse events, response rates, predictors for responders, and treatment persistence.

**Results:**

In the entire cohort, the MMD changes from baseline at 3, 6, 12, and 24 months were  − 7.2 ± 4.7,  − 8.1 ± 6.3,  − 8.4 ± 5.1, and  − 9.6 ± 6.0 days, respectively (*p* < 0.001). After 3, 6, 12, and 24 months, the ≥ 50% response rates were 57.0%, 63.6%, 63.5%, and 69.0%, respectively. The MIDAS score significantly decreased in the total sample and the EM and CM groups. No significant difference in efficacy was found between the monthly and quarterly dosing groups. Adverse events, mainly injection site reactions, occurred in 13.3% of the patients, and 2.4% of the participants discontinued treatment due to side effects. There were different clinical backgrounds between non-responders, and early and ultra-late responders, including psychiatric complications, medication overuse headache, and pulsatile headache. The treatment continuation rates at 12, 18, and 24 months were 73.5%, 65.4%, and 58.0%, respectively, with higher persistence in patients who received quarterly dosing than in those who received monthly dosing (*p* < 0.001).

**Conclusion:**

Fremanezumab is effective and well tolerated for long-term migraine prophylaxis.

## Introduction

Migraine affects more than one billion people worldwide [1], and despite advances in diagnosis and treatment, it remains the second most common cause of disability worldwide [1, 2]. Not only headache attacks but also interictal symptoms, including stigma, depressive symptoms, and perceived anxiety related to unpredictable attacks, can impair the quality of life of patients [3], and comprehensive management of patients by effective prophylaxis is warranted.

Monoclonal antibodies (mAbs) that target calcitonin gene-related peptide (CGRP), which is involved in the pathophysiology of migraine, are now available, and their efficacy and safety have been demonstrated in multiple randomized controlled trials [4]; favorable results from real-world studies are now accumulating [5].

Fremanezumab is a humanized immunoglobulin G2 monoclonal antibody with a half-life of 31 days that targets the alpha and beta isoforms of CGRP [6], and in Japan, it was approved for migraine prophylaxis for adult migraine patients in 2021. Fremanezumab is administered at a dose of 225 mg monthly or 675 mg quarterly, depending on patient preference and physician recommendation. The efficacy of fremanezumab has been reported in several real-world studies, including our previous study involving a 6-month follow-up [7]. However, most of the evidence is based on 3–6 months of treatment with fremanezumab for migraine patients with different backgrounds [8–11], with a maximum of 48–52 weeks of treatment [12–14]. Moreover, the results of a 12-month interim analysis of a multicenter, prospective, observational, phase 4 study of fremanezumab have just been reported [15].

Patients with severe or frequent migraines who have difficulty visiting a hospital every month may prefer quarterly fremanezumab dosing to monthly fremanezumab dosing. However, most fremanezumab studies from the U.S. and Europe include only a small percentage of the quarterly dosing group [8, 12, 13], and further evidence on quarterly dosing would be clinically useful. Therefore, in the present study, we extended our previous 6-month fremanezumab study in migraine patients and provided results on its efficacy, safety, and tolerability over 2 years, with a median follow-up of 14.0 months.

## Methods

### Study design

We conducted a retrospective, observational, single-center cohort study of long-term fremanezumab treatment in migraine patients. Our study was approved by the Ethics Committee of Dokkyo Medical University Hospital. In accordance with the Declaration of Helsinki, all participating patients were briefed about this observational study at an outpatient clinic and given the opportunity to opt out of participation in the study. Owing to the retrospective, observational nature of this study, the Ethics Committee determined that it was unnecessary for patients to sign an informed consent form.

In our study, the primary endpoint was the change in the number of monthly migraine days (MMDs) from baseline to months 1 and 24. The secondary endpoints included adverse events over 24 months; 3-, 6-, 12-, 18-, and 24-month response rates; changes in the Migraine Disability Assessment (MIDAS) score; and treatment persistence. Additionally, subanalysis by monthly or quarterly dosing groups was performed.

### Patients

**Fig. 1:**
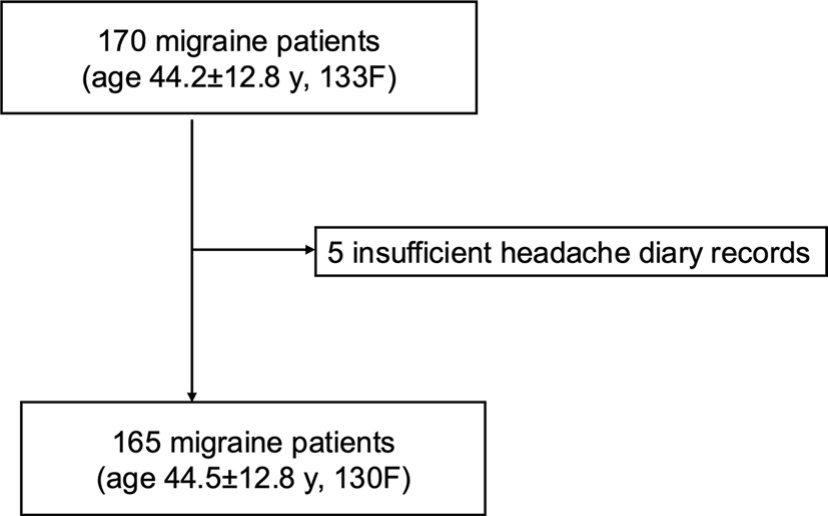
Flowchart of the study

Among patients who visited our headache outpatient clinic between April 2022 and January 2025, patients with EM or CM who were at least 18 years of age and who received at least one dose of fremanezumab were eligible for this study. The flowchart of our study is illustrated in Fig. 1. Out of 170 migraine patients, after excluding 5 patients whose headache diary records were insufficient, 165 patients (age = 44.5 ± 12.8 y, 130 females) were ultimately included in this study. The patients had received treatment with at least one or more headache prophylaxis medications prior to the initiation of either monthly (225 mg) or quarterly (675 mg) doses of fremanezumab.

### Diagnosis of migraine

Migraine with or without aura was diagnosed by neurologists after exclusion of secondary headache by head magnetic resonance imaging according to the criteria of the International Classification of Headache, 3rd edition (ICHD-3) [16]. Chronic migraine was defined as having headaches lasting at least 15 days/month for at least 3 months, during which migraine features were present for at least 8 days/month, and EM was defined as having headaches occurring for 4 to 14 days/month. Medication overuse headache (MOH) was diagnosed according to the ICHD-3 [16].

### Clinical assessments

Clinical data on migraine duration, migraine characteristics, comorbidities, aura, accompanying symptoms, and history of prophylactic medications were obtained from medical records. The number of MMD from baseline to 1–24 months after fremanezumab treatment was obtained from paper headache diaries. The MIDAS scores were obtained before and 9–12 months after fremanezumab treatment.

On the basis of the headache diary, the percent reduction in MMDs from baseline after fremanezumab treatment was calculated, and response rates at 1, 3, 6, 12, 18, and 24 months were classified into groups of < 30%, 30–49%, 50–74%, and 75–100%, respectively. Early responders were defined as patients who achieved a 50% or greater reduction from baseline in MMDs at month 3, late responders were defined as patients who achieved a 50% or greater MMD reduction at months 4–6, and ultra-late responders were defined as patients who achieved a 50% or greater MMD reduction after month 6 according to a previous study [17]. Clinical factors associated with early, late and ultra-late responders were compared.

### Statistical analysis

Given the real-world setting of this study, no sample size calculations were performed. Mann‒Whitney U tests or Student's t tests were used for comparisons of continuous variables as appropriate, and chi‒square tests were used for comparisons of categorical variables. One-way analysis of variance (ANOVA) or the Kruskal‒Wallis test followed by Bonferroni’s post-hoc test, and the chi‒square test or Fisher‒Freeman‒Halton exact test were performed to compare the four groups. The Wilcoxon signed rank test was used to compare MIDAS scores before and after fremanezumab treatment. A generalized linear mixed-effects model (GLMM) followed by Bonferroni's post-hoc test was used to test for a significant difference from baseline to each month for MMDs after treatment with fremanezumab. The effects of two different fremanezumab doses, monthly and quarterly, on MMD reduction were analyzed by two-way ANOVA using the GLMM followed by a global test.

Kaplan‒Meier survival curves were used to estimate treatment persistence rates. Log-rank tests were used for comparisons between two groups. Thirteen patients who switched from monthly to quarterly dosing were excluded from the Kaplan‒Meier analysis of the monthly vs. quarterly dosing groups.

A two-tailed *p* value < 0.05 was considered to indicate statistical significance. IBM SPSS Statistics version 28 (IBM SPSS, Tokyo, Japan) and GraphPad Prism for Mac (version 8; GraphPad Software, San Diego, USA) were used for the statistical analyses. GraphPad Prism for Mac (version 8; GraphPad Software, San Diego, USA) and Microsoft Excel version 16.18 were used to generate the figures.

## Results

The patient background information for this study is shown in Table 1. Among a total of 165 patients, there were 79 cases of EM (47 with monthly fremanezumab dosing and 32 with quarterly fremanezumab dosing) and 86 cases of CM (65 with monthly fremanezumab dosing and 21 with quarterly fremanezumab dosing). The mean number of previous preventive medication classes was 2.3 ± 1.4, and 45 patients (27.3%) had previously used another CGRP mAb. The total sample had a mean of 16.5 ± 6.8 MMDs; moreover, the CM group had a significantly greater mean number of MMDs (21.7 ± 5.0) than the EM group did (10.9 ± 2.8). Compared with the EM group, the CM group had a 34.9% greater prevalence of MOH, with less aura and pulsatile headache and more comorbidities. Initially, 112 patients (47 EM and 65 CM) were on monthly dosing, but 13 of them (5 EM and 8 CM) changed to quarterly dosing after an average of 1.4 ± 0.7 months. The use of migraine prophylaxis tended to be greater in the CM group than in the EM group (Table 2).

**Table 1:** Baseline characteristics of patients with migraine

	Total	EM	CM	p value
n (M/F)	165 (35/130)	79 (20/59)	86 (15/71)	0.216
Initial fremanezumab dosing, monthly/quarterly	112/53	47/32	65/21	**0.027**
Age, years	44.5 ± 12.8	43.3 ± 12.8	45.5 ± 12.8	0.282
Body mass index (kg/m^2^)	22.4 ± 3.7	22.2 ± 3.9	22.6 ± 3.5	0.527
Migraine with aura, n (%)	27 (16.4)	18 (22.8)	9 (10.6)	**0.035**
Medication overuse headache, n (%)	30 (18.2)	0 (0.0)	30 (34.9)	** < 0.001**
Disease duration, years	25.2 ± 12.4	23.4 ± 12.8	26.8 ± 11.9	0.083
Pain location, n (%)				
Unilateral	107 (64.8)	52 (65.8)	55 (64.0)	0.802
Bilateral	119 (72.1)	54 (68.4)	65 (75.6)	0.301
Pain characteristics, n (%)				
Pulsating	147 (89.1)	76 (96.2)	71 (82.6)	**0.005**
Pressing	109 (66.1)	49 (62.0)	60 (69.8)	0.294
Others	4 (2.4)	2 (2.5)	2 (2.3)	0.931
Accompanying symptoms, n (%)				
Photophobia	132 (80.0)	65 (82.3)	67 (77.9)	0.483
Phonophobia	130 (78.8)	61 (77.2)	69 (80.2)	0.636
Osmophobia	83 (50.3)	43 (54.4)	40 (46.5)	0.309
Nausea	148 (89.7)	76 (96.2)	72 (83.7)	**0.008**
Allodynia	40 (24.2)	19 (24.1)	21 (24.4)	0.956
Number of previous preventive medication classes, n (%)	2.3 ± 1.4	1.8 ± 1.0	2.7 ± 1.7	** < 0.001**
Baseline MMDs	16.5 ± 6.8	10.9 ± 2.8	21.7 ± 5.0	** < 0.001**
MIDAS score	27.4 ± 17.0	19.9 ± 22.4	32.0 ± 28.8	**0.027**
Comorbidities, n (%)	104 (63.0)	41 (51.9)	63 (73.3)	**0.005**
Psychiatric diseases, n (%)	28 (17.0)	12 (15.2)	16 (18.6)	0.559

*EM* episodic migraine, *CM* chronic migraine, *M* male, *F* female, *MMDs* monthly migraine days; MIDAS = Migraine Disability Assessment

Mann‒Whitney U tests or Student's t tests and chi‒square tests were used. Statistically significant values (*p* < 0.05) are indicated in bold

**Table 2:** Prophylactic medication use in patients with migraine

	Total	EM	CM	*p* value
Migraine prophylactic medication use, n (%)	57 (34.5)	22 (27.8)	35 (40.7)	0.083
Calcium channel blockers, n (%)	17 (29.8)	7 (31.8)	10 (28.6)	0.794
Beta blockers, n (%)	6 (10.5)	4 (18.2)	2 (5.7)	0.192
Topiramate, n (%)	3 (5.3)	0 (0.0)	3 (8.6)	0.276
Valproic acid, n (%)	7(12.3)	6 (27.3)	1 (2.9)	**0.011**
Amitriptyline, n (%)	21 (36.8)	6 (27.3)	15 (42.9)	0.235
Others, n (%)	5 (8.8)	1 (4.5)	4 (11.4)	0.639

*EM* episodic migraine, *CM* chronic migraine

The Chi-square test or Fisher’s exact test were used

Statistically significant values (*p* < 0.05) are indicated in bold

### Efficacy

#### Total cohort

**Fig. 2:**
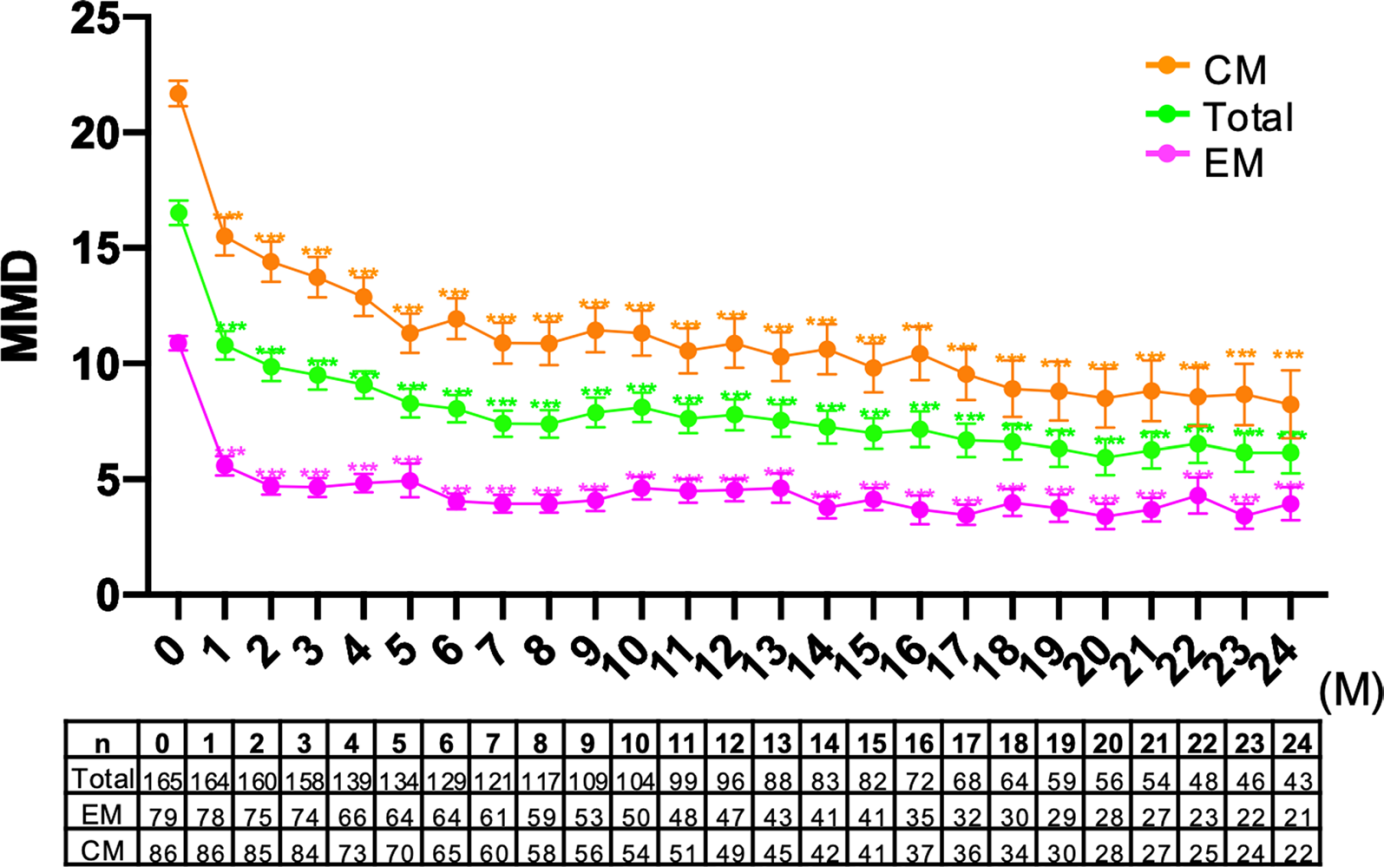
Changes in monthly migraine days from baseline to 24 months after fremanezumab treatment in total, EM and CM patients. ***, *p* < 0.001. A generalized mixed-effects model with repeated measures and Bonferroni correction was used. *EM* episodic migraine, *CM* chronic migraine

**Fig. 3:**
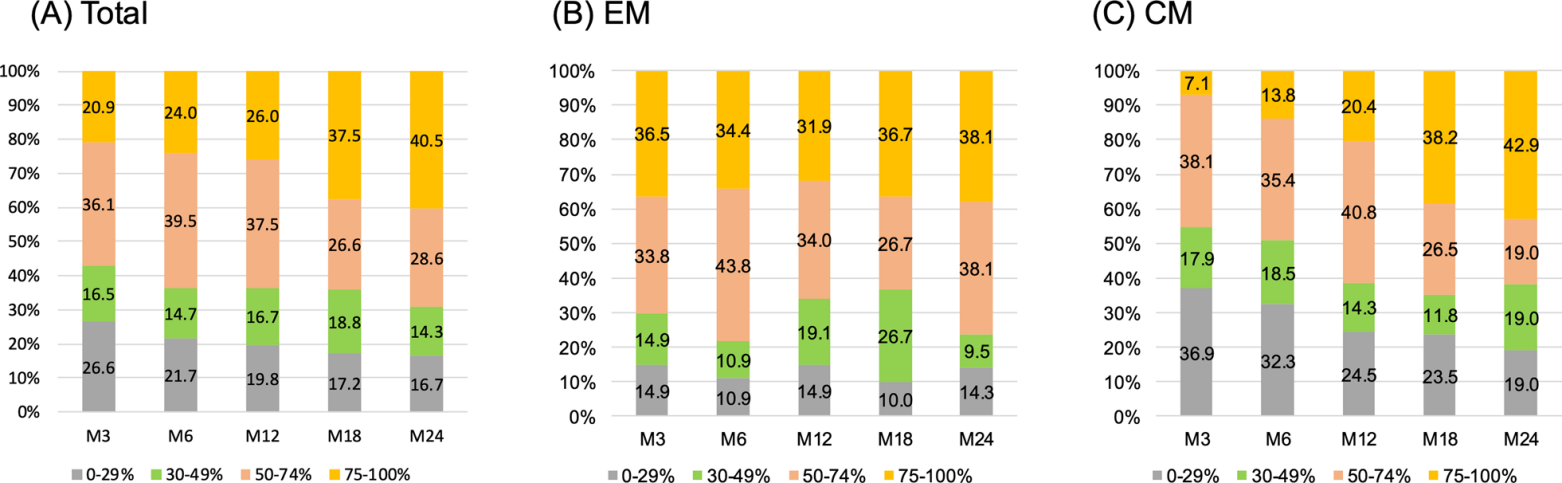
Proportion of responders after fremanezumab treatment. *EM* episodic migraine, *CM* chronic migraine

The MIDAS scores improved significantly from baseline after fremanezumab treatment (27.4 ± 17.0 vs. 19.4 ± 24.3, *p* < 0.001). In the entire cohort, MMDs decreased by − 7.2 ± 4.7 days, − 8.1 ± 6.3 days, − 8.4 ± 5.1 days, − 9.1 ± 6.0 days, and -9.6 ± 6.0 days at months 3, 6, 12, 18, and 24, respectively, after fremanezumab treatment. Repeated-measures ANOVA with GLMMs and Bonferroni’s post hoc test revealed significant reductions in MMDs compared with those at baseline after 1 to 24 months of treatment with fremanezumab (Fig. 2). After 3, 6, 12, 18, and 24 months, the ≥ 50% response rates were 57.0%, 63.6%, 63.5%, 64.1%, and 69.0%, respectively, and the ≥ 75% response rates were 20.9%, 24.0%, 26.0%, 37.5%, and 40.5%, respectively (Fig. 3).

#### EM group

The MIDAS scores significantly decreased from baseline after fremanezumab treatment (19.9 ± 22.3 vs. 12.0 ± 15.4, *p* < 0.001). In the EM group, the baseline MMDs were 10.9 ± 2.8 days. After fremanezumab treatment, the MMDs decreased by − 6.3 ± 3.2 days, − 6.8 ± 3.2 days, − 6.4 ± 3.2 days, − 6.3 ± 3.7 days, and − 6.6 ± 3.7 days at months 3, 6, 12, 18, and 24, respectively (Fig. 2). GLMM analysis followed by Bonferroni’s post hoc test revealed that MMD values were significantly lower after 1–24 months of fremanezumab treatment than at baseline. In the EM group, after 3, 6, 12, 18, and 24 months, the ≥ 50% response rates were 70.3%, 78.1%, 66.0%, 63.3%, and 76.2%, respectively, and the ≥ 75% response rates were 36.5%, 34.4%, 31.9%, 36.7%, and 38.1%, respectively (Fig. 3).

#### CM group

There was a significant reduction in the MIDAS score from baseline after fremanezumab treatment (32.0 ± 28.8 vs. 23.9 ± 27.5, *p* < 0.001). The CM group had 21.7 ± 5.0 MMDs at baseline. After fremanezumab treatment, the MMDs decreased by − 7.9 ± 5.6 days, − 9.2 ± 5.8 days, − 10.2 ± 5.8 days, − 12.0 ± 6.5 days, and − 12.4 ± 6.6 days at months 3, 6, 12, 18, and 24, respectively (Fig. 2). MMDs were significantly reduced throughout months 1–24 of fremanezumab treatment compared to those at baseline, according to the Bonferroni's post hoc test following analysis using GLMM. In the CM group, after 3, 6, 12, 18, and 24 months, the ≥ 50% response rates were 45.2%, 49.2%, 61.2%, 64.7%, and 61.9%, respectively, and the ≥ 75% response rates were 7.1%, 13.8%, 20.4%, 38.2%, and 42.9%, respectively (Fig. 3).

### Monthly versus quarterly dosing

**Fig. 4:**
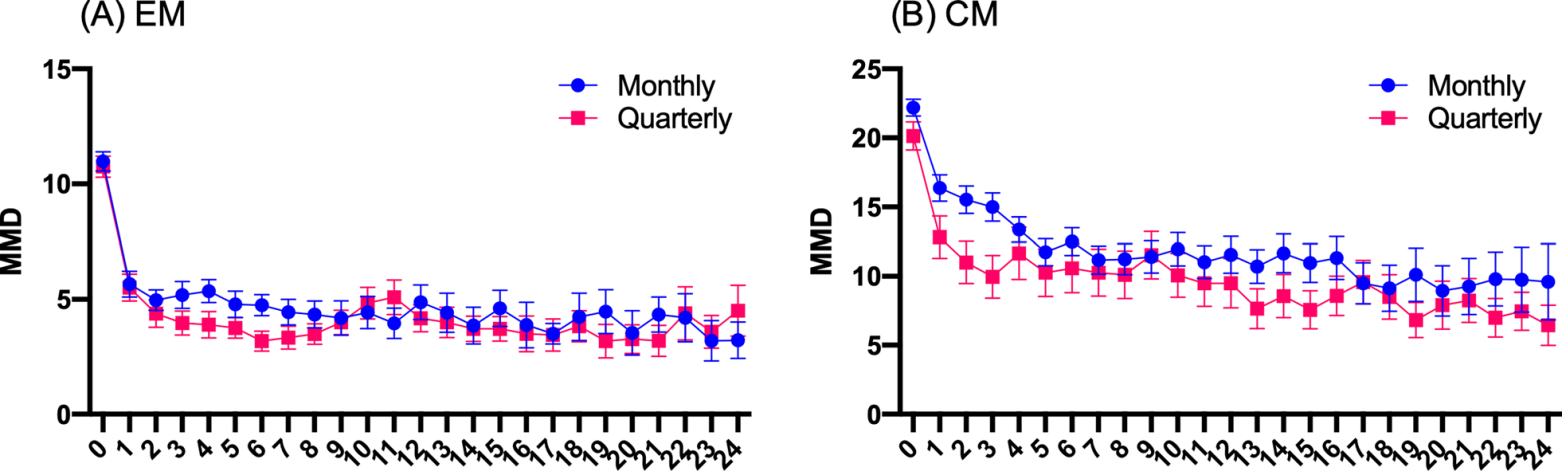
Mean number of monthly migraine days at baseline and after fremanezumab treatment in EM and CM patients receiving monthly and quarterly dosing. *EM* episodic migraine, *CM* chronic migraine

In the EM group, there was a significant difference in time (F = 28.12, *p* < 0.001), but there were no differences between the dose groups (F = 1.133, *p* = 0.291) or interaction between time and dose (F = 1.014, *p* = 0.444) (Fig. 4). Similarly, in the CM group, there was a significant difference in time (F = 19.88, *p* < 0.001) but no difference between the dosing groups (F = 3.925, *p* = 0.051), and there was no interaction between time and dose (F = 0.975, *p* = 0.497).

### Comparison of background factors among nonresponders and responders

Table 3 shows the clinical background factors for nonresponders, early responders, late responders, and ultra-late responders. Nonresponders had increased baseline MMDs, CM and MOH. Early responders received a greater proportion of monthly dosing, had a greater proportion of EM and pulsatile headache, and had fewer baseline MMDs. The ultra-late responders had no psychiatric complications, a lower prevalence of MOH (8.3%), and a greater prevalence of pulsatile headache (91.7%).

**Table 3:** Clinical features of migraine patients who were nonresponders and responders

	Nonresponders	Early responders	Late responders	Ultra-late responders	*p* value
n (M/F)	33 (2/31)	89 (23/66)	28 (6/22)	12 (2/10)	0.092
EM/CM	8/25	52/37	12/16	4/8	**0.006**
Initial fremanezumab dosing, monthly/quarterly	29/4	51/38	21/7	8/4	**0.008**
Age, years	43.5 ± 13.5	45.4 ± 13.1	43.4 ± 12.4	44.5 ± 9.8	0.839
Body mass index (kg/m^2^)	21.5 ± 3.9	22.3 ± 3.7	23.1 ± 4.1	21.8 ± 2.3	0.406
Migraine with aura, n (%)	6 (18.8)	18 (20.2)	1 (3.6)	2 (16.7)	0.186
Medication overuse headache, n (%)	12 (36.4%)	9 (10.1)	8 (28.6)	1 (8.3)	**0.003**
Disease duration, years	24.5 ± 12.0	25.7 ± 13.2	24.1 ± 11.7	26.5 ± 8.6	0.890
Pain location, n (%)					
Unilateral	18 (54.5)	58 (65.2)	19 (67.9)	9 (75.0)	0.572
Bilateral	25 (75.8)	64 (71.9)	20 (71.4)	8 (66.7)	0.938
Pain characteristics, n (%)					
Pulsating	26 (78.8)	85 (95.5)	22 (78.6)	11 (91.7)	**0.008**
Pressing	23 (69.7)	58 (65.2)	20 (71.4)	7 (58.3)	0.812
Others	2 (6.1)	0 (0.0)	1 (3.6)	0 (0.0)	0.098
Accompanying symptoms, n (%)					
Photophobia	24 (72.7)	71 (79.8)	24 (85.7)	10 (83.3)	0.679
Phonophobia	25 (75.8)	67 (75.3)	24 (85.7)	11 (91.7)	0.499
Osmophobia	16 (48.5)	43 (48.3)	14 (50.0)	8 (66.7)	0.690
Nausea	29 (87.9)	82 (92.1)	23 (82.1)	11 (91.7)	0.432
Allodynia	7 (21.2)	19 (21.3)	8 (28.6)	5 (41.7)	0.388
Number of previous preventive medication classes, n (%)	2.9 ± 1.6	2.1 ± 1.4	2.1 ± 1.2	2.3 ± 1.4	0.203
Baseline MMDs	20.3 ± 7.8	14.4 ± 5.6¶	18.1 ± 6.6	19.4 ± 6.9	** < 0.001**
MIDAS score*	32.4 ± 22.1	21.6 ± 22.4	34.8 ± 37.1	44.3 ± 45.6	0.204
Comorbidities, n (%)	24 (72.7)	55 (61.8)	18 (64.3)	6 (50.0)	0.515
Psychiatric diseases, n (%)	10 (30.3)	10 (11.2)	8 (28.6)	0 (0.0)	**0.01**

*EM* episodic migraine, *CM* chronic migraine, *M* male, *F* female, *MMDs* monthly migraine days, *MIDAS* Migraine Disability Assessment

One-way analysis of variance or the Kruskal‒Wallis test followed by Bonferroni test and the chi‒square test or Fisher‒Freeman‒Halton exact test were performed to compare the four groups

Statistically significant values (*p* < 0.05) are indicated in bold. ¶*p* < 0.05 vs. nonresponders

*Available for 83 patients

### Safety

Overall, 22 (13.3%) patients experienced adverse reactions: 21 (12.7%) experienced injection site reactions, and 1 (0.6%) experienced constipation. Four patients (2.4%) discontinued treatment or switched to other CGRP mAbs because of adverse reactions.

### Treatment persistence

**Fig. 5:**
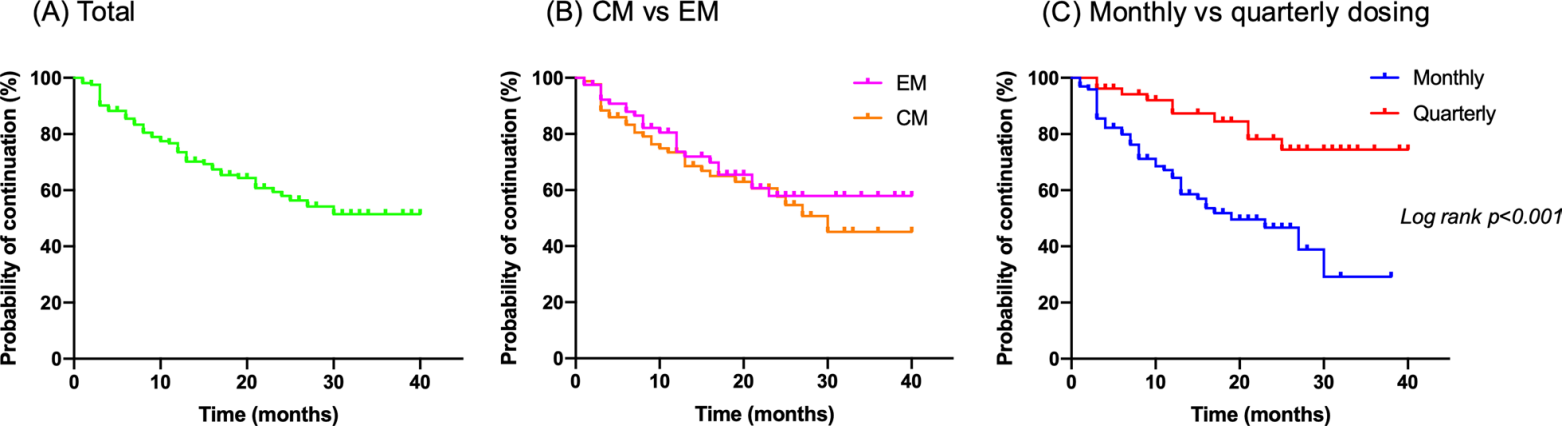
Kaplan‒Meier curve for the continuation of fremanezumab. *EM* episodic migraine, *CM* chronic migraine

The median follow-up duration of fremanezumab treatment was 14.0 months (range, 1–40 months). The treatment continuation rate was 73.5% at 12 months, 65.4% at 18 months, and 58.0% at 24 months (Fig. 5A). Overall, 97 patients (58.8%) continued treatment, 16 (9.7%) discontinued treatment due to headache improvement, 27 (16.4%) discontinued treatment due to ineffectiveness or adverse reactions, and 24 (14.5%) switched to other CGRP mAbs. In the CM and EM groups, the treatment continuation rates were similar: 80.5% and 73.4% at 12 months, 65.5% and 65.1% at 18 months, and 57.9% and 57.7% at 24 months, respectively (Fig. 5B). In the monthly and quarterly dosing groups, the treatment continuation rates were 64.4% and 87.4% at 12 months, 51.9% and 84.5% at 18 months, and 46.7% and 78.2% at 24 months, respectively. The treatment continuation rate was significantly higher in the quarterly dosing group than in the monthly dosing group (*p* < 0.001; Fig. 5C).

## Discussion

In the present study, we evaluated the efficacy and safety of fremanezumab in patients with EM or CM who were resistant to at least one preventive treatment over a longer observation period than our previous studies of 6 to 9 months [7, 18], providing the longest observation period (over 24 months) among real-world studies. In the entire cohort, the number of MMDs, the primary endpoint, decreased significantly after treatment with fremanezumab. In a recent observational study of fremanezumab quarterly dosing that included 28 migraine patients (15 CM and 13 EM), the mean change in MMD from baseline was − 2.2 at 3 months, − 1.8 at 12 months, and − 1.6 at 2 years [19], whereas in our study the MMD decrease was greater over the 2-year period. In our study, MMD reduction was greater in the CM group than in the EM group, whereas the percentage of responders was greater in the EM group than in the CM group. There was also a significant improvement in headache-related disability, as measured by the MIDAS, after fremanezumab initiation. Furthermore, the efficacy of fremanezumab monthly and quarterly dosing was comparable over 24 months, as was a 6-month observational study of fremanezumab [7].

A prospective, multicenter study in Italy evaluated the efficacy of monthly doses of fremanezumab for 52 weeks in 90 patients with high-frequency EM and CM [13]. That study revealed a significant reduction in the number of MMDs and acute medication use, with 76.5% of patients having a 50% ≥ response rate at 12 months. The treatment was well tolerated, with eight patients (9.6%) experiencing at least one adverse event and only one patient (1.1%) discontinuing treatment due to adverse reactions. In another Italian multicenter study including patients with high-frequency EM and CM treated with fremanezumab (127 monthly and 3 quarterly), 75.5% of migraine patients had at least a 50% reduction in MMD at 12 months, with a mean reduction in MMD of 14.5 days [12]. Mild and transient adverse events were observed in 7.8% of the patients. Although the MMD response rate and MMD reduction were milder in our study than in those studies at 12 months [12, 13], the safety was comparable. In our study, 13.3% of patients experienced side effects, mostly injection site reactions, and only 2.4% discontinued or switched to other CGRP mAbs because of side effects. The difference in the efficacy of fremanezumab may have been affected by the difference in patient background, which included 27.3% of previous CGRP mAb users in our study. In contrast, a 52-week, randomized, open-label, parallel-group study of fremanezumab revealed adverse reactions in 84–90% of patients; at 12 months, a ≥ 50% MMD reduction was reported in 23–68% of patients, with a reduction in MMDs of 1–8 days [20, 21]. The difference in safety and efficacy between our study and ours may have been influenced by differences in the study setting (randomized controlled trials vs. real-world studies).

We investigated the persistence of treatment with fremanezumab and reported that it was well tolerated with an overall average of 14 months. In our study, 32.1% of patients received quarterly dosing of fremanezumab, and treatment persistence was greater for quarterly dosing than for monthly dosing, which may reflect that quarterly dosing is a more convenient treatment approach that fits the patient's lifestyle better than monthly dosing is. In a survey regarding the fremanezumab dosing regimen, 69.2% preferred quarterly fremanezumab over monthly [22]. Patients can reduce their frequency of hospital visits with fremanezumab home self-injection, but they may still have fear/anxiety about self-injection and the burden of monthly injections [23]. The reasons for preferring quarterly dosing over monthly dosing may include a reduced injection burden and improved treatment compliance [24], and the fact that migraine is more common in the productive age group makes quarterly dosing of fremanezumab an important option for improving patients' quality of life.

In the present study, we compared clinical background factors between nonresponders and early, late, and ultra-late responders. As in several previous studies, nonresponders had greater baseline MMDs and CM and MOH prevalence rates [7, 25]. In this study, ultra-late responders did not invariably have the same background factors as late responders did. Patients with a pulsatile headache without MOH or psychiatric comorbidities could become ultra-responders if they continue treatment for more than 6 months.

The study limitations included the lack of a control prophylactic treatment group for the fremanezumab treatment group and the incomplete follow-up for some of the included migraine patients. Unilateral autonomic symptoms, which have been reported to be associated with ultra-late responders [17], were not assessed in this study.

## Conclusion

Our study investigated the efficacy and safety of fremanezumab over 24 months at a single center. Treatment persistence for more than 2 years with a median follow-up of 14.0 months was achieved. Quarterly dosing is as effective as monthly dosing and may prolong treatment continuity.

## Data Availability

The datasets from this study are available from the corresponding author upon reasonable request.

## Abbreviations

EM: Episodic migraine
CM: Chronic migraine
M: Male
F: Female
MMDs: Monthly migraine days
MIDAS: Migraine disability assessment
MOH: Medication overuse headache
